# Detecting peripersonal space: The promising role of ultrasonics

**DOI:** 10.1002/brb3.1085

**Published:** 2018-08-09

**Authors:** Antonino Chillura, Antonino Naro, Fabrizio Ciappina, Alessia Bramanti, Paola Lauria, Placido Bramanti, Rocco Salvatore Calabrò

**Affiliations:** ^1^ IRCCS centro Neurolesi “Bonino‐Pulejo” Messina Italy

**Keywords:** cerebral blood flow, motor control, peripersonal space, social cognition, transcranial ultrasound

## Abstract

**Introduction:**

The approach of an external stimulus to the peripersonal space (PPS) modifies some physiological measures, including the cerebral blood flow (CBF) in the supplementary motor area and premotor cortex. CBF measurement may be useful to assess brain activations when producing specific motor responses, likely mediated by cortical and subcortical neural circuits.

**Methods:**

This study investigated PPS in 15 healthy humans by characterizing the hemodynamic responses (pulsatility index, PI; and heart rate, HR) related to different directions of movements of individual's hand toward and backward his/her own face, so to perturb PPS).

**Results:**

We observed that the CBF and HR were enhanced more when the stimulated hand was inside the PPS of the face in the passive and active condition than when the hand was outside the PPS and during motor imagery task.

**Conclusions:**

These results suggest that the modulation of PPS‐related brain responses depends on specific sensory‐motor integration processes related to the location and the final position of a target in the PPS. We may thus propose TCD as a rapid and easy approach to get information concerning brain responses related to stimuli approaching the PPS. Understanding the modulations of brain activations during tasks targeting PPS can help to understand the results of psychophysical and behavioral trials and to plan patient‐tailored cognitive rehabilitative training.

## 1 INTRODUCTION

Movement planning and execution to grasp or avoid objects require the exact prediction and monitoring of the positions and movements of body parts. To this end, several sensory‐motor integration processes concerning the “body representations” (including the position and dimension of body parts) and the space around the body (i.e., the peripersonal space and PPS) have to occur (De Vignemont, 2010). PPS representation is made of multisensory, body part‐centered reference frames, so to elaborate automated or finalized motor responses to the surrounding stimuli (Bourgeois & Coello, 2012). Wide frontoparietal networks allow integrating multisensory information within the PPS in monkeys and humans (Bremmer et al., 2001; Di Pellegrino, Ladavas, & Farné, 1997; Graziano, Taylor, & Moore, 2002; Làdavas & Serino, 2008; Makin, Holmes, & Zohary, 2007; di Pellegrino & Làdavas, 2015; Rizzolatti, Fadiga, Fogassi, & Gallese, 1997; Rizzolatti, Scandolara, Matelli, & Gentilucci, 1981; Sambo & Forster, 2009; Spence, Pavani, Maravita, & Holmes, 2008). Such a PPS representation has also a motor function, that is, it links together the instantaneous multisensory representation of PPS with the pertinent potential motor acts (Bourgeois & Coello, 2012; Cooke, Taylor, Moore, & Graziano, 2003; di Pellegrino & Làdavas, 2015).

Nonetheless, the neurophysiology of PPS‐related cortical processing is partially understood. Innovative electrophysiological and neuroimaging approaches have highlighted the involvement of large‐scale cortical networks within frontal and parietal cortices by exploring cerebral hemodynamics through cerebral blood flow (CBF), metabolic rate, and oxygenation (Bartolo et al., 2014; Cléry, Guipponi, Wardak, & Ben Hamed, 2015; di Pellegrino & Làdavas, 2015). Altogether, these measures showed significant changes in response to cognitive and motor tasks related to PPS (Brozzoli, Makin, Cardinali, Holmes, & Farnè, 2012; Coello & Fischer, 2015; Costantini, Ambrosini, Scorolli, & Borghi, 2011; Longo & Lourenco, 2007; Makin et al., 2007; Maranesi, Bonini, & Fogassi, 2014). The role and functionalities of defensive PPS have been extensively explored using motor tasks aimed at perturbing PPS (Bisio et al., 2017; Sambo, Forster, Williams, & Iannetti, 2012; Sambo & Iannetti, 2013; Sambo, Liang, Cruccu, & Iannetti, 2012). About that, the hand blink reflex (HBR), that is, the electromyographic activity recorded from both orbicularis oculi muscles elicited by the electrical stimulation of the median nerve at the wrist, was elicited when the hand was positioned at different hand‐to‐face distances. It has been demonstrated that the magnitude of the HBR increased with the proximity of the stimulated hand to the face, irrespective of the position of the arm, of the head, and whether the eyes were open or closed. Moreover, the magnitude of HBR was greater when the hand approaching the face was stimulated near than far the face and when the participant expected to receive the electric stimulus to the hand when it was close to the face. Finally, HBR was sensitive to movement planning, that is, it was sensitive to the predictive role of motor system, which can anticipate the consequence of the movement (Bisio et al., 2017). Altogether, these results provide evidence that the brain exerts a fine somatotopical and cognitive tuning of the excitability of brainstem circuits subserving the HBR, whose strength is adjusted in a purposeful manner depending on the motor scenario.

The present work aimed at studying PPS in healthy humans by characterizing the hemodynamic responses (pulsatility index, PI; and heart rate, HR) related to different directions of movements of individual's hand toward and backward his/her own face (so to perturb PPS). We chose Transcranial Doppler ultrasound (TCD) to monitor CBF changes because of its low cost, excellent temporal resolution and sensitivity to CBF changes within the circle of Willis and the large intracranial vessels, as shown in a variety of neurologic disorders (Purkayastha & Sorond, 2012). The variables we measured are commonly used to estimate cerebrovascular responsiveness to various stimuli (Watt, Burnfield, Truemper, Buster, & Bashford, 2012). In particular, one can estimate the distal cerebrovascular resistance by measuring the PI, which depends on multiple hemodynamic variables including the metabolic changes during cognitive tasks (thus being possible to indirectly estimate changes in brain metabolism) (D'Andrea et al., 2016; Demarin & Morovic, 2014; Kenney et al., 2016; Kim & Lee, 2015; Purkayastha & Sorond, 2012; Wolf, 2015).

To perturb PPS, we employed three different motor task in analogy to previous electrophysiological studies (Bisio et al., 2017; Sambo & Iannetti, 2013; Sambo, Forster, et al., 2012; Sambo, Liang, et al., 2012). We tested the effects on CBF of hand movements toward (CBF increase) and backward the face (CBF decrease) performed actively and passively (i.e., by an examiner). We expected a modulation of CBF depending on hand‐to‐face distance and direction, particularly within the territories depending on the middle cerebral artery, given that only afferent (visual and proprioceptive) information was available to estimate the final state of the system (i.e., the position of the hand with respect to the face) in such motor conditions. We also estimated the effects of mental activity processes, that is, motor imagery, on CBF. We expected a modulation of CBF depending on hand‐to‐face distance and direction, particularly within the territories depending on the anterior cerebral artery, given that only efferent (intentional) information was available to predict the consequences of the movement in such motor condition. To demonstrate the specificity of CBF modulation when approaching PPS, participants were provided with two control motor tasks, in which the participant observed a motor scenario targeting participant's PPS or was passively subjected to a movement outside the PPS. We opted for such motor tasks as both movement observation and actions targeting extrapersonal space entrain brain networks, and thus evoke CBF changes, which are different from those found when PPS is entrained (Caspers, Zilles, Laird, & Eickhoff, 2010; Cléry et al., 2015; Cross, Kraemer, Hamilton, Kelley, & Grafton, 2009). Altogether, such CBF changes would indicate that a dynamic prediction (target positions with respect to the PPS and its related consequences) of PPS‐centered motor scenario is run by the sensorimotor system and is based on the integration of feedforward and sensory feedbacks, thus allowing to plan, and eventually execute, purposeful motor responses within PPS (Bisio et al., 2017).

## 2 MATERIALS AND METHODS

### 2.1 Participants

Fifteen subjects (eight females and seven males, mean age 31 ± 8 years, range 25–49) without a history of neurological, psychiatric, or orthopedic disease were selected for this study. All of them were right‐handed and naïve to the purpose of the study. None of them was undergoing medical treatment or practiced vasoactive drugs in the 24 hr preceding the assessment. All procedures were approved by the Ethics Committee of our Institute, and all subjects gave written informed consent. The research followed the tenets of the Declaration of Helsinki.

The sample size estimate was based on extrapolations from previous studies examining the effects of motor tasks targeting the PPS (Marra et al., 2018; Naro et al., 2018). Accordingly, we used the effect size (0.9) of the endpoint (PI change) for calculations. Power was set at 80%, with alpha at 5%. Using a relatively conservative estimation, 15 subjects would be required to detect a statistically significant PI change.

### 2.2 Experimental design

**Figure 1:**
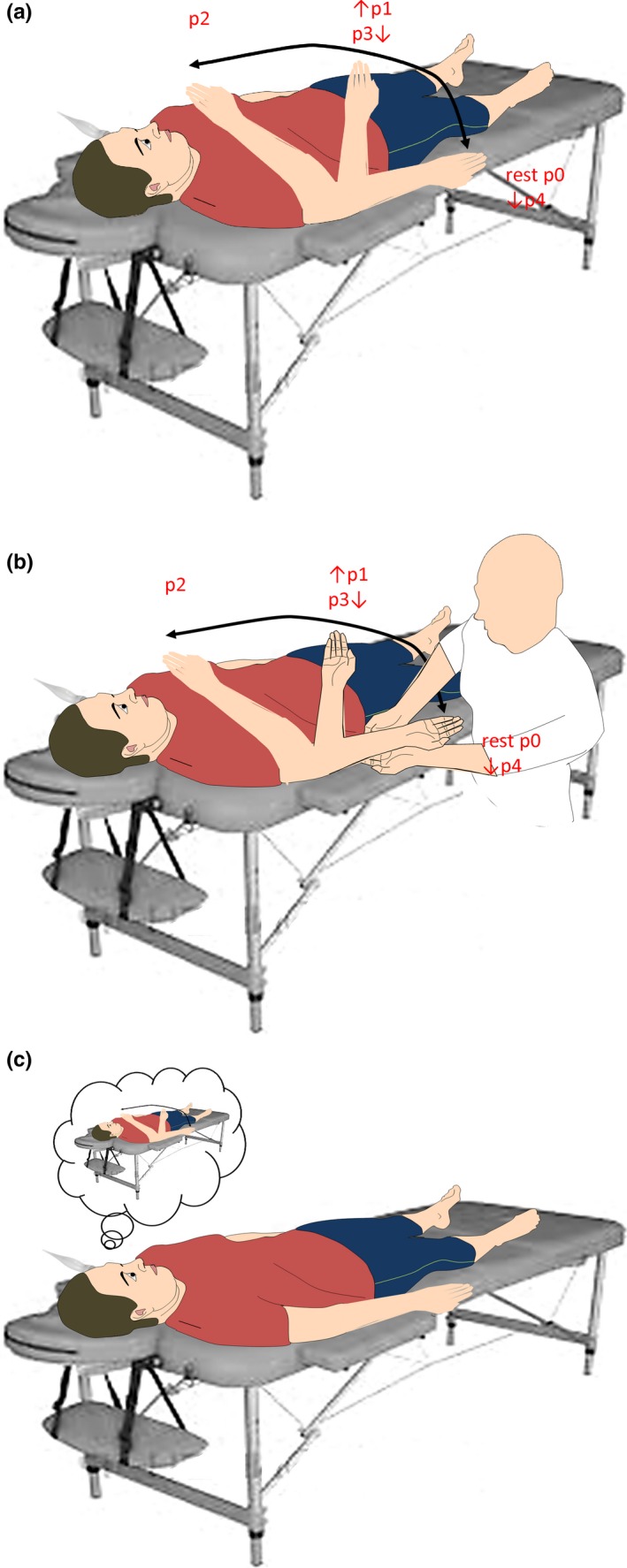
Summary of experimental procedures in the active (a), passive (b), and motor imaging (c) condition

The participant was lying supine on the stretcher in a quiet and semi‐darkened room, keeping the eyes opened, with the right upper limb lying along the trunk, with the palm facing up. A headrest minimized head movements. At first, we performed TCD in resting condition (*p0*_*rest*_), that is, while the participant stayed still. Then, participants were provided with three different motor tasks that were carried out in distinct sessions, with an intersession break of 30 min, and in a random order. The task consisted of sequential flexion‐extension movements of the upper limb (Figure 1a,b,c): First, the participants had to flex the right forearm from the resting position (*p0 *=* *180°) to 90° (*p1*) and then up to 10° toward the face (*p2*). After that, they had to extend the upper limb from *p2* to *p3* (equal to *p1*) and then to *p4* (equal to *p0*). Each movement onset was synchronized individually with participant's HR, so that the recording time was matched between conditions. The speed of right upper limb movements within and between movements had to be kept as constant as possible.

Such sequence of movements was performed actively (Figure 1a), passively (Figure 1b) and in motor imagery (Figure 1c). In the active condition, the participant was instructed to move immediately after hearing an acoustic cue that was set on individual's HR, every ten beats. In the passive task, an experimenter passively mobilized the right upper limb in the positions mentioned above. Participants were invited to keep their upper limb completely relaxed (Figure 1b). The onset of the movement performed by the experimenter was synchronized with subject's HR (by an acoustic cue that was set on individual's HR, every ten beats). During movement (passive and active), participants and experimenters had to keep as constant as possible the speed of right upper limb movements. In the imaginary task (Figure 1c), the individuals had to imagine the right upper limb movement across the above‐mentioned positions, while keeping the upper limb at rest (*p0*_*rest*_). The onset of the imaginary movement was synchronized with subject's HR by an acoustic cue (every ten beats). Moreover, participants were asked to press a button with the left hand at the end of each imaginary movement, whose duration was measured. Given that pushing a button can activate the same neural circuits involved in motor imagery (including supplementary motor area and premotor cortex), thus potentially inducing modifications in the CBF, we excluded a period of 2 s before pushing the button from the analysis (Lu, Mamun, & Chau, 2014; Vingerhoets & Stroobant, 1999).

Finally, in two distinct control experiments, we evaluated the effects on CBF of (a) the hand of the experimenter going forward and backward the face of the participant in a “passive condition” fashion (namely “movement observation”), through the above‐mentioned hand positions (p0, p1, p2, p3, and p4); and (b) the upper limb of the patient performing an abduction movement from the trunk (p0), up to 45 (p1) and 90 deg (p2) in parallel to the stretcher, and then an adduction toward the trunk (45 deg, p3, and 0 deg, p4). Even in such control experiments, the onset of the movements (performed by the experimenter—control experiment i‐ or the subject—control experiment ii) was synchronized with individual's HR by an acoustic cue (every ten beats). The speed of right upper limb movements within and between movements had to be kept as constant as possible.

PI and HR changes at each hand position were recorded from middle (MCA), anterior (ACA), and posterior cerebral artery (PCA). Each experiment was performed twice, and the average of PI and HR values was analyzed.

### 2.3 Transcranial Doppler ultrasound evaluation

Transcranial ultrasound was performed using a conventional ultrasound system color‐coded with a 2–5 MHz phased‐array transducer (iU22 Philips Healthcare Solutions; Bothell, WA, US). The examination was performed through the left temporal acoustic bone window (Krejza et al., 2007), with the transducer located anterior to the tragus and upward the zygomatic arch. The peak systolic velocity (PSV), end‐diastolic velocity (EDV), and mean velocity (Vm) were measured for MCA, ACA, and PCA. Age‐ and gender‐corrected PI were calculated from each vessel according to the formula. These measures were obtained in p0rest and p0, so to establish the regulatory parameters that were used for subsequent measurements at each of the following positions during passive, active, and motor imaging task.

### 2.4 Statistical analysis

PI and HR at rest (p0rest) between the explored arteries were compared using *t* tests. Then, PI and HR modulation were analyzed using an ANOVA with the factors *artery* (three levels: ACA, MCA, and PCA), *hand‐position* (four levels: *p0→p1*,* p1→p2*,* p2→p3*, and *p3→p4*), and *task* (three levels: passive, active, and motor imaging). The factors *artery* and *task* were not used for the parameter HR and the control experiments (i), respectively. The Greenhouse–Geisser method was used if necessary to correct for nonsphericity. A *p‐*value of <0.05 was considered significant. Conditional on a significant *F*‐value, post hoc *t* tests (Bonferroni corrected) were performed to explore the strength of main effects and interactions. All data are given as mean ± *SD*.

### 2.5 Ethical approval

All procedures performed in studies involving human participants were in accordance with the ethical standards of the institutional and/or national research committee and with the 1964 Helsinki declaration and its later amendments or comparable ethical standards.

## 3 RESULTS

In resting condition, PI and HR were similar between the three main arteries we explored (MCA = 0.76 ± 0.07; ACA = 0.74 ± 0.05; and PCA = 0.75 ± 0.05; HR = 70 ± 4 bpm; all *p*‐values >0.2).

**Figure 2:**
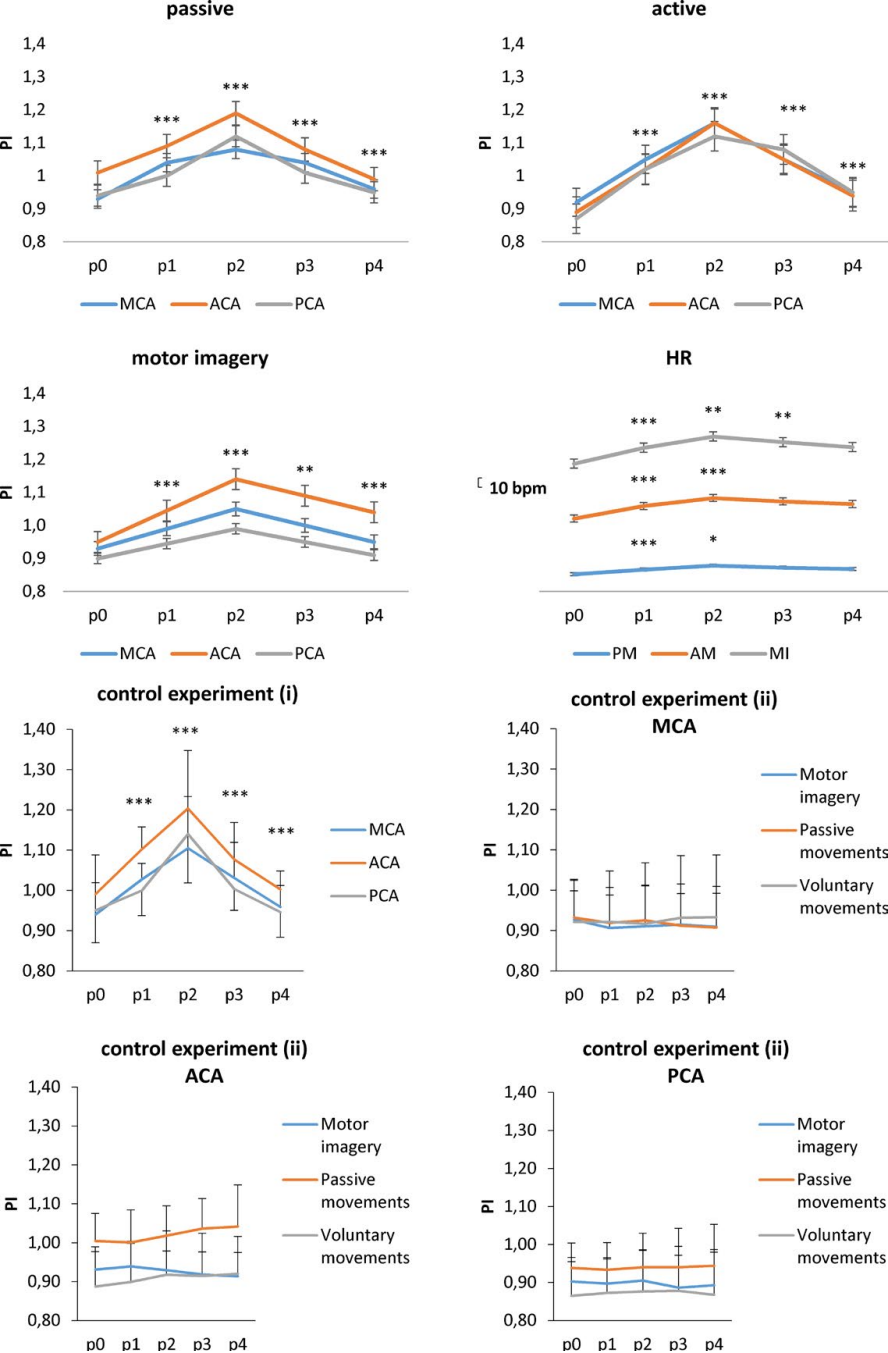
The mean PI and HR changes during passive (PM) and active movements (AM), motor imagery (MI), and control experiment (i) and (ii) of upper limb across the different positions (p), from anterior (ACA), middle (MCA), and posterior cerebral artery (PCA). Vertical error bars refer to *SD*. * indicates the significance of each PI and HR change at each position as compared to the previous one (Bonferroni corrected *p*‐value ***p < 0.001, **p = 0.001, *p* < 0.01)

Data analysis indicated that hand‐to‐face distance significantly influenced PI magnitude, as revealed by the significant *hand‐position* effect (*F*_(3,42)_ = 95, *p* < 0.001). In general, PI increased when going toward the face (*p0→p1* and *p1→p2*, in which we found the highest PI values) and decreased when going backward (*p2→p3* and *p3→p4*, with *p1 *≈* p3* and *p0 *≈* p4*) (Figure 2). Moreover, PI changes depended on the motor task (*hand‐position *× *task* interaction *F*_(6,84)_ = 5.3, *p* < 0.001). In fact, the overall PI magnitude changes were greater in the active than passive and motor imagery task (Figure 2). The *artery × hand‐position *× *task* (*p* = 0.3), *hand‐position *× *artery* (*p* = 0.6), and *artery* (*p* = 0.5) interactions and effects were nonsignificant. No difference of PI between the arteries was appreciable (Figure 2).

**Figure 3:**
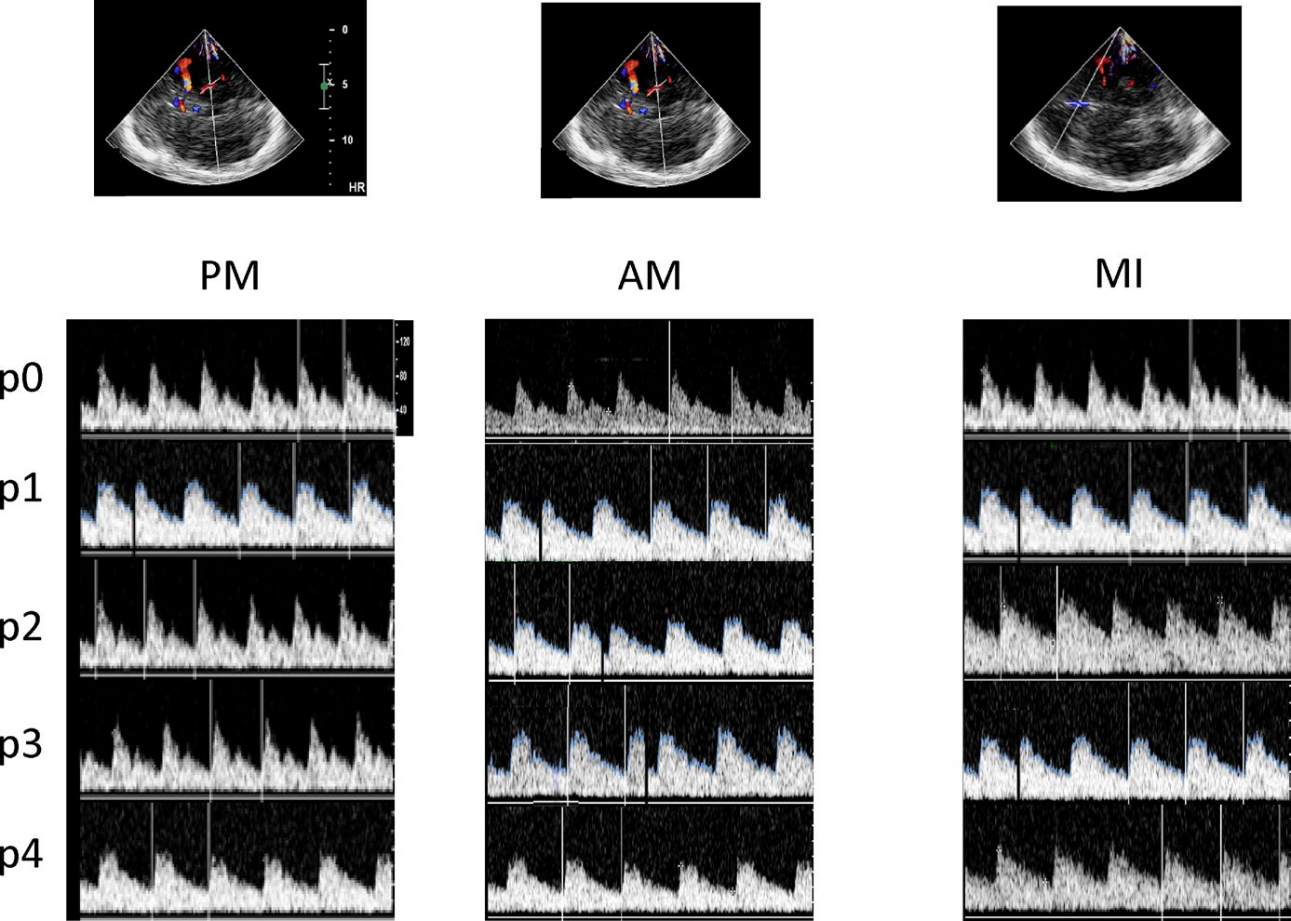
Some examples of the most evident TCD changes from middle cerebral artery during passive (PM) and active (AM) movements of upper limb across the different positions (p) and from anterior cerebral artery during motor imagery (MI)

We then performed two‐way repeated‐measures ANOVA for the hand‐to‐face distances across the average PI, so to investigate the hand‐to‐face distance course of the effects of the experimental factors *task* and *artery* across the whole PI response. The *task *× *artery* main interaction was not significant (*p* = 0.1), as the hand‐to‐face distance significantly changed the PI in both passive and active tasks without artery differences (*hand‐position *× *artery* interaction in the passive task *p* = 0.6; *hand‐position* effect for each artery *p* < 0.001; *hand‐position *× *artery* interaction in the active task *p* = 0.9; *hand‐position* effect for each artery *p* < 0.001). Instead, motor imagery task modified PI differently between the arteries (*hand‐position *× *artery* interaction *F*_(6,84)_ = 3, *p* = 0.004; *hand‐position* effect for ACA *p* < 0.001, for MCA *p* = 0.3, for PCA *p* = 0.2). In fact, a PI modulation was detectable only in the ACA (PI increase at *p0→p2* and decrease at *p2→p4*) (Figure 2). Representative examples of TCD images for passive, active, and motor imaging tasks are provided in Figure 3.

Heart rate was analyzed using two‐way repeated measure ANOVA with the factors *hand‐position* and *task*. Data analysis indicated that hand‐to‐face distance significantly influenced HR, as revealed by the significant *hand‐position* effect (*F*_(3,42)_ = 5.8, *p* = 0.002). In general, HR increased when going toward the face (*p0→p1* and *p1→p2*, in which we found the highest PI values) and decreased when going backward (*p2→p3* and *p3→p4*, with *p1 *≈* p3* and *p0 *≈* p4*) (Figure 2). Moreover, PI changes depended on the motor task (*hand‐position *× *task* interaction *F*_(6,84)_ = 3.3, *p* = 0.001). In fact, the HR average values were higher during motor imagery as compared to the active and passive condition (Figure 2) and limitedly to *p0→p1* and *p1→p2* in the active and motor imagery task (Figure 2).

The effects of movement observation (control experiment (i)) on PI were analyzed using two‐way repeated measure ANOVA with the factors *hand‐position* and *artery*. We found a significant *hand‐position* effect on PI increase‐decrease (*F*_(4,56)_ = 27, *p* < 0.001) (as found for the main experiments) (Figure 2), with neither significant difference between arteries (*artery* effect *p* > 0.05) nor *hand‐position *× *artery* interaction (*p* > 0.05).

Finally, the effects of abduction–adduction movement (control experiment (ii)) on PI were analyzed using three‐way repeated measure ANOVA with the factors *hand‐position*,* task*, and *artery*. We found no significant main effects of interactions (all *p* > 0.05) (Figure 2).

## 4 DISCUSSION

To the best of our knowledge, this is the first study assessing CBF changes induced by motor tasks targeting PPS. According to previous electrophysiological studies (Bisio et al., 2017; Sambo & Iannetti, 2013; Sambo, Forster, et al., 2012; Sambo, Liang, et al., 2012), CBF increased when the hand approached the PPS face, whereas it decreased when the hand receded from the face. Such a PI modulation involved all three arteries explored during both the passive and active conditions, that is, it occurred when visual and proprioceptive information were available to the subject. Contrariwise, PI modulation was more focused during motor imaging (i.e., within ACA), when only efferent (intentional) information was available to the subject. PI changed also during control experiment (i) (i.e., experimenter's hand moving toward and backward subject's face), thus confirming that fTCD gives reliable information concerning brain response related to stimuli approaching the PPS, and excluding that PI modulation may be simply related to a generic movement execution rather than to a real correlation between movement execution and PPS entrainment. On the other hand, movements performed by the subjects outside the PPS did not influence PI (control experiment (ii)), thus confirming that fTCD gives different information whether a stimulus approaches PPS or extrapersonal space.

Such wide CBF modulations are in keeping with previous works indicating the activation of frontoparietal networks where the integration of sensory feedforward and feedback signals (including tactile, visual, and auditory stimuli provided near the body) related to the motor task demand and the surrounding environment occurs (Bremmer et al., 2001; Duhamel, Bremmer, Ben Hamed, & Graf, 1997; Graziano & Cooke, 2006; Graziano, Yap, & Gross, 1994; Gritsenko, Yakovenko, & Kalaska, 2009; Makin et al., 2007; Medendorp, 2011; Rizzolatti et al., 1981; Serino, Canzoneri, & Avenanti, 2011). These networks are functionally separated from that controlling the extrapersonal space, as suggested by control experiment (ii) results.

Through these processes, it is possible to estimate the forthcoming position of a target within the PPS and its related consequences by a motoric point of view (Blanke, Slater, & Serino, 2015). This is possible through an integrated, highly dynamic system controlling both visual and tactile inputs within PPS, which is more sensitive to the final position where the hand is expected to be than the actual position where the hand is (Bisio et al., 2017). In fact, PI increased more in the *p1→p2*, that is, with the hand approaching the PPS face, and *p2→p3* transition, that is, with the hand leaving the PPS face, than the other positions. The greater amount of PI changes in such hand positions may depend on the fact that the visual experience of the moving upper limb, as in the *p1→p2* and *p2→p3* transitions, potentiates the processing of feel‐touch experience (Làdavas, Zeloni, & Farnè, 1998). On the other hand, motor (intentional outflow) and sensory (inflow) information are sufficient to tune CBF when the visual experience is not available, as in the *p0→p1* and *p3→p4* transitions (Blakemore, Wolpert, & Frith, 2002; Haggard, 2005; Wolpert & Flanagan, 2001; Wolpert, Ghahramani, & Jordan, 1995). Differently from the study of Bisio et al. (2017), we did not observe a different PI modulation between *p1* (moving toward the face) and *p3* (moving away from the face). This discrepancy may depend on the different nature of these approaches. In fact, the modulation of HBR magnitude has a temporal resolution in the millisecond order, whereas the CBF regulation takes place in the order of seconds.

In parallel, the extent of PI changes also differed between the motor tasks, maybe depending on the available amount of sensory information. In fact, motor imagery task (where only intentional outflow was present) yielded a focal (i.e., within ACA) and milder CBF increase. This could depend on the fact that the individuals internally regulated the extent of their movements, thus reducing the needs of an extensive PPS modulation (as in the passive and active motor tasks). Additionally, the type of motor task could differently activate the top‐down control originating from motor and associative cortical areas, thus changing the needs of CBF increase (Miwa, Nohara, Hotta, Shimo, & Amemiya, 1998; Sambo, Forster, et al., 2012; Sambo, Liang, et al., 2012).

Pulsatility index changes were paralleled by equivalent HR modulations (i.e., an increase when going toward the face—from *p0* to *p2*—and a decrease when moving backward—from *p2* to *p4*). One could concern that this companion variation may simply reflect a general autonomic activity sustained by subcortical network activation, as observed in a typical startle response (in that, a stimulus approaching toward the face), being thus unrelated to PPS perturbation. Nonetheless, the motor tasks we employed require at least one or more among physical, psychological, and mental effort to be executed, depending on the hand‐to‐face distance (for purposeful or reflex actions to be eventually planned and executed), the comfort distance (for potential interactions with the experimenter within participant's PPS), judgments toward stimuli while during passive or active task—namely participant's or experimenter's hand (Ferri, Ardizzi, Ambrosecchia, & Gallese, 2013; Iachini, Coello, Frassinetti, & Ruggiero, 2014; Proulx, Todorov, Taylor Aiken, & de Sousa, 2016). Therefore, HR changes may represent a specific marker of PPS perturbation.

In keeping with the presence of another individual within the participant's PPS, one could hypothesize the involvement of social issues of the PPS, given that social environment and the presence and interaction with others shape the PPS representation, and PPS mediates the interaction with other targets (objects/individuals) (Fossataro, Sambo, Garbarini, & Iannetti, 2016; Iachini et al., 2014; Pellencin, Paladino, Herbelin, & Serino, 2017; Quesque et al., 2017; Teneggi, Canzoneri, di Pellegrino, & Serino, 2013). Beyond social interaction issues, CBF during passive mobilization was likely triggered by both the tactile inputs to the individual by the experimenter and the proprioceptive feedback related to the arm position toward the face. Therefore, we can actually only speculate on the social issues of the PPS, as we should have performed a control experiment with another person sitting near the participant in the active condition, and this should be addressed in future studies.

### 4.1 Limitations

fTCD measures CBFV rather than absolute cerebral blood flow. An estimation of the latter can be made if the diameter of the insonated vessel remains constant (Salinet, Panerai, & Robinson, 2012; Salinet, Robinson, & Panerai, 2013), but there is not sufficient data to demonstrate this issue in our work. fTCD has an interesting temporal resolution, but the spatial resolution is unfortunately low so that we cannot be precise on spatialized cerebral hemodynamics. A possible contribution from peripheral covariates (including beat‐to‐beat arterial blood pressure, HR, PaCO_2_, breath‐by‐breath end‐tidal CO_2_, and the neural activation stimulus represented by the go signal) could lead to the inaccurate assessment of CBFV, particularly during motor imagery (Salinet et al., 2012, 2013). However, blood pressure and HR (which were continuously monitored) did not significantly correlate with PI values and changed according to a typical waxing–waning pattern.

One could be concerned about the nearly linear transition of CBF and HR measures we found when the hand moved toward and backward the face, whereas previous works (e.g., Teneggi et al., 2013) have reported that brain responses show a subject‐dependent sigmoid transition across hand positions. This discrepancy may depend on the methodological approach we used, given that fTCD offers a continuous data sampling with high temporal resolution, as compared to the discrete sampling of reaction time tasks.

One could acknowledge some confounds associated with the presence of the arm into PPS or imaging it there, including enhanced attention for a close by object in PPS, dynamic visual cues due to the looming of a visual stimulus into PPS (speed, size changes), and proprioceptive cues related to arm position with respect to the body (Cléry et al., 2018; Sambo & Iannetti, 2013). However, the specific PI modulation we found within the different motor tasks makes unlike this concern.

## 5 CONCLUSIONS

Our data indicate that the generation of brain response related to stimuli approaching the PPS (of the face, at least) is continuously shaped by the predictive motor system (that elaborates sensory inflow), also depending on the evaluation of other people's behavior during social interactions. The brain responses evoked during PPS perturbation could depend on top‐down regulation processes supported by frontoparietal networks. We may propose TCD as a rapid and easy approach to furnish new information concerning brain responses when perturbing PPS. The understanding of CBF changes related to PPS perturbation can contribute to understanding the results of psychophysical and behavioral trials in either healthy or neurologic/neuropsychiatry conditions (e.g., poststroke neglect) (Cuadrado, Egido, Gonzalez‐Gutierrez, & Varela‐de‐Seijas, 1999; Halligan & Marshall, 1991; Holt et al., 2015; Riestra & Barrett, 2013; Silvestrini, Cupini, Placidi, Diomedi, & Bernardi, 1998).

## CONFLICT OF INTEREST

None of the authors has conflict of interests.

## INFORMED CONSENT

Informed consent was obtained from all individual participants included in the study.
